# Supplementary material for: Analysis of Association between Breastfeeding and Vegetable or Fruit Intake in Later Childhood in a Population-Based Observational Study
Source: Int J Environ Res Public Health. 2020 May 26;17(11):3755. doi: 10.3390/ijerph17113755 (PMC7312394; doi:10.3390/ijerph17113755)
Supplement: Supplementary file 1 [file ijerph-17-03755-s001.pdf]

International Journal of
Environmental Research and Public Health

MDPI

Analysis of Association between Breastfeeding and Vegetable or Fruit Intake in Later Childhood in a Population-based Observational Study

Jadwiga Hamulka*, Monika A. Zielinska, Marta Jeruszka-Bielak, Magdalena Górnicka, Dominika Głąbska, Dominika Guzek, Monika Hoffmann and Krystyna Gutkowska

Supplementary Materials

Table S1. The associations between breastfeeding duration and vegetable and fruit consumption for second breakfast.

Group	Breastfeeding duration	Vegetable consumption			Fruit consumption		
		**Model 1** [1] OR [4] (95% CI [5])	**Model 2** [2] aOR [6] (95% CI)	**Model 3** [3] aOR (95% CI)	**Model 1** [1] OR [4] (95% CI [5])	**Model 2** [2] aOR [6] (95% CI)	**Model 3** [3] aOR (95% CI)
**Total**	No breastfeeding		Ref. 1			Ref. 1	
	0.2–3 months	1.54 (0.79–3.17)	1.65 (0.84–3.24)	1.63 (0.83–3.22)	1.24 (0.76–2.04)	1.28 (0.78–2.68)	1.27 (0.82–2.11)
	4 –6 months	1.33 (0.68 -2.70)	1.39 (0.70–2.76)	1.37 (0.69–2.73)	1.26 (0.77–2.06)	1.29 (0.78–3.15)	1.28 (0.95–2.12)
	7 –12 months	1.36 (0.70–2.70)	1.24 (0.63–2.47)	1.26 (0.63–2.49)	1.12 (0.69–1.84)	1.10 (0.66–2.42)	1.10 (0.74–1.82)
	>12 months	1.29 (0.60–3.30)	1.29 (0.59–2.82)	1.32 (0.60–2.91)	0.97 (0.49–1.55)	1.00 (0.50–1.98)	1.00 (0.50–1.62)
**Girls**	No breastfeeding		Ref. 1			Ref. 1	
	0.2–3 months	0.95 (0.39–2.33)	1.05 (0.41–2.65)	1.06 (0.42–2.70)	1.99 (0.95–4.14)	2.22 (1.04–4.73)*	2.23 (1.05–4.76)*
	4–6 months	0.88 (0.36–1.15)	0.89 (0.35–2.26)	0.90 (0.35–2.29)	1.47 (0.71–3.03)	1.56 (0.74–3.30)	1.56 (0.74–3.31)
	7–12 months	1.23 (0.53–2.87)	1.15 (0.48–2.78)	1.20 (0.50–2.89)	1.07 (0.53–2.18)	1.04 (0.50–2.16)	1.06 (0.51–2.20)
	>12 months	0.95 (0.34–2.69)	0.92 (0.32–2.66)	0.93 (0.32–2.71)	1.15 (0.49–2.67)	1.22 (0.52–2.87)	1.23 (0.52–2.90)
**Boys**	No breastfeeding		Ref. 1			Ref. 1	
	0.2–3 months	2.72 (0.96–7.74)	2.70 (0.93–7.82)	2.57 (0.88–7.48)	1.08 (0.43–1.66)	1.18 (0.41–1.61)	1.15 (0.40–1.59)
	4–6 months	2.22 (0.77–6.41)	2.30 (0.78–6.80)	2.23 (0.75–6.61)	1.10 (0.56–2.15)	1.05 (0.53–2.08)	1.03 (0.52–2.05)
	7–12 months	1.48 (0.48–4.51)	1.38 (0.44–4.33)	1.32 (0.42–4.18)	1.19 (0.60–2.37)	1.20 (0.59–2.43)	1.19 (0.59–2.42)
	>12 months	1.96 (0.59 –6.46)	1.95 (0.58 –6.55)	2.02 (0.60–6.83)	1.09 (0.31–2.52)	1.08 (0.31–1.60)	1.07 (0.32–1.60)

[1] Model 1 – unadjusted model; [2] Model 2 – model adjusted for children age, BMI centile (and gender in total group), maternal education, employment status and family economic situation; [3] Model 3 – Model 2 adjusted for EU–28 average PKB region; [4] OR – odds ratio; [5] CI – confidence interval; [6] aOR – adjusted odds ratio; * – $p \leq 0.05$.

Table S2. The associations between breastfeeding duration and vegetable and fruit consumption between meals.

Group	Breastfeeding duration	Vegetable consumption			Fruit consumption		
		**Model 1** [1] OR [4] (95% CI [5])	**Model 2** [2] aOR [6] (95% CI)	**Model 3** [3] aOR (95% CI)	**Model 1** [1] OR [4] (95% CI [5])	**Model 2** [2] aOR [6] (95% CI)	**Model 3** [3] aOR (95% CI)
**Total**	No breastfeeding		Ref. 1			Ref. 1	
	0.2–3 months	1.44 (0.36–5.71)	1.46 (0.36–5.87)	1.44 (0.36–5.78)	1.02 (0.62–1.68)	1.07 (0.64–1.79)	1.08 (0.65–1.81)
	4 –6 months	1.79 (0.17–3.58)	1.86 (0.18–3.79)	1.83 (0.18–3.85)	1.31 (0.79–2.16)	1.38 (0.82–2.32)	1.39 (0.83–2.33)
	7 –12 months	1.38 (0.35–5.47)	1.45 (0.36–5.91)	1.41 (0.35–5.75)	1.95 (0.58–1.55)	1.89 (0.53–1.48)	1.89 (0.53–1.49)
	>12 months	2.85 (0.71–5.38)	3.20 (0.79–5.98)	3.23 (0.80–6.11)	1.88 (0.49–1.57)	1.87 (0.49–1.57)	0.87 (0.48–1.56)
**Girls**	No breastfeeding		Ref. 1			Ref. 1	
	0.2–3 months	1.30 (0.23–7.36)	1.36 (0.22–8.59)	1.37 (0.22–8.66)	1.88 (0.40–2.93)	1.94 (0.41–2.11)	1.94 (0.42–2.14)
	4–6 months	1.29 (0.03–3.30)	1.23 (0.02–2.80)	1.24 (0.24–2.93)	0.91 (0.41–2.98)	1.98 (0.43–2.21)	1.94 (0.41–2.13)
	7–12 months	1.07 (0.19–6.06)	1.02 (0.14–5.82)	0.92 (0.14–5.84)	1.59 (0.28–1.25)	1.55 (0.25–2.20)	1.53 (0.24–2.17)
	>12 months	1.21 (0.16–9.00)	1.33 (0.16–10.88)	1.33 (0.16–10.92)	1.76 (0.31–1.88)	1.81 (0.32–2.04)	1.81 (0.32–2.04)
**Boys**	No breastfeeding		Ref. 1			Ref. 1	
	0.2–3 months	1.79 (0.18–7.62)	1.55 (0.15–5.93)	1.56 (0.15–6.32)	1.17 (0.60–2.30)	1.18 (0.60–2.34)	1.19 (0.60–2.37)
	4–6 months	1.79 (0.18–7.62)	1.44 (0.13–5.43)	1.51 (0.14–6.21)	1.75 (0.89–3.46)	1.80 (0.90–3.60)	1.82 (0.91–3.65)
	7–12 months	1.96 (0.20–4.76)	1.80 (0.17–6.89)	1.85 (0.17–9.64)	1.37 (0.69–2.720	1.30 (0.64–2.63)	1.30 (0.64–2.64)
	>12 months	6.16 (0.69–9.38)	6.77 (0.74–8.03)	7.08 (0.77–9.47)	1.01 (0.45–2.16)	1.02 (0.42–2.03)	1.02 (0.42–2.03)

[1] Model 1–unadjusted model; [2] Model 2–model adjusted for children age, BMI centile (and gender in total group), maternal education, employment status and family economic situation; [3] Model 3–Model 2 adjusted for EU–28 average PKB region; [4] OR–odds ratio; [5] CI–confidence interval; [6] aOR–adjusted odds ratio.
